# The manipulation of photon blockade via Newtonian gravity

**DOI:** 10.1038/s41598-024-64206-1

**Published:** 2024-06-10

**Authors:** Zhen Li, Wang-Jun Lu, Yun-Lan Zuo

**Affiliations:** 1 Department of Physics, Shaoyang University, Shaoyang, 422099 China; 2 Department of Physics, Zhejiang Institute of Modern Physics, Zhejiang University, Hangzhou, 310027 China; 3 Key Laboratory of Low-Dimensional Quantum Structures and Quantum Control of Ministry of Education, Department of Physics and Synergetic Innovation Center for Quantum Effects and Applications, Hunan Normal University, Changsha, 410081 China

**Keywords:** Single photons and quantum effects, Theoretical physics

## Abstract

We theoretically investigate the model of a quadratically coupled optomechanical system with a Newtonian gravitational potential in the weak-driving regime, where the optical cavity is driven by an external laser. The steady state of the whole system is treated in the framework of a few-photon subspace. We find that the conventional single-photon blockade, nonstandard types of single-photon blockade, two-photon blockade, and photon-induced tunneling can be induced by gravity when the quadratic optomechanical coupling strength remains constant. Moreover, we find that gravitational potential energy can compensate for the lack of quadratic optomechanical coupling for observation photon blockade. In particular, the photon stream with super-Poissonian distribution can be converted into a sub-Poissonian, antibunching photon stream by changing the driving detuning when the gravitational potential energy is included. These results show that the gravity has potential for realizing the manipulation of photon blockade in a quadratically coupled optomechanical system.

The investigation of light-matter interactions is a key issue in quantum optics and quantum information science for several decades. The key prerequisite for investigating the characteristics of light-matter interactions is that the generation and manipulation of photons. Thus, we have to prepare a source of photon streams. Photon blockade (PB)^1–3^ is one of the ways in which this source can be accomplished. The single-photon blockade is that the subsequent photons will be prevented from entering the cavity when there is already a photon in the cavity due to the strong optical nonlinearity in the system^4–6^. This optical nonlinearity means that the energy levels of the optical field are anharmonic. The optical nonlinearity can be induced by the interaction between the cavity photons and other subsystems^7–12^. Obviously, the PB realizes that an incident photon stream with Poissonian distribution will be transformed into a sub-Poissonian distribution, antibunching photon stream. This suggests that the PB can convert the input of a classical optical field to that of a quantum optical field. The PB effect has been observed experimentally for the first time in an optical cavity coupled to a single trapped atom^1^. It has also been experimentally demonstrated in other systems^13–16^. In addition, the PB effect has been studied in other systems, and our understanding of it has advanced enormously in recent years^17–26^.

The study of optomechanical systems, where the mechanical oscillator is coupled to one or more driven cavity modes through radiation-pressure coupling, has opened the door to a lot of applications in quantum physics^27,28^. The optomechanical systems have recently made some progress in quantum state preparation^29–32^, entanglement of mechanical systems^33–37^, signal processing^38,39^, quantum sensing^40,41^, topological physics^42,43^, and small-scale thermodynamics^44–46^. One of the important milestones in the study of optomechanical systems is to enable the sensitivity of weak force measurements to reach or even extensively surpass the standard quantum limit^47–53^. The key prerequisite for this milestone in optomechanical systems is that an external force on the mechanical oscillator changes the equilibrium position of the mechanical oscillator. Thus, the external force can indirectly modulate the resonant frequency of the cavity via the optomechanical coupling^54^. Then, a large difference in the cavity output fields will result in a highly amplified homodyne signal^55^. Obviously, the optomechanical system is very sensitive to external forces. From another perspective, these are also sufficient to show that an external weak force can have a large effect on the optomechanical system. One question that arises naturally is whether the properties of the optomechanical system can be manipulated by external weak forces.

Motivated by the above discussions, we plan to study the manipulation of photon blockade using a quadratic optomechanical system with gravitational coupling. The quadratic optomechanical system consists of a high-finesse Fabry-Pérot cavity and a flexible dielectric membrane. The quadratic optomechanical coupling is fabricated between the cavity and the membrane, where the membrane is precisely placed at a node or antinode of the standing wave in the cavity, when the cavity is driven by the external laser^56–58^. Gravity has no effect on the quadratic optomechanical coupling in a horizontally placed optomechanical system, since the direction of light interaction with the membrane is horizontal. However, the gravity will affects the quadratic optomechanical coupling when we rotate the optomechanical device by an angle from the vertical axis due to the equilibrium position of the membrane will be changed^59–62^. Then, the gravitational coupling is similar to the direct current (DC) phonon drive. This allows the properties of the quadratic optomechanical system to be manipulated by external gravity.

The remainder of this paper is organized as follows. Firstly, we introduce the physical model and provide the full quadratic optomechanical Hamiltonian including the gravitational potential. Then, the eigenvalues and eigenstates of the whole system, apart from the external optical driving, can be obtained by the squeezing and displacement transformation. After this, the steady-state of the system can be obtained within the few-photon subspace in the weak-driving regime when an anti-Hermitian term is phenomenologically added to the Hamiltonian. Secondly, we numerically calculated the second-order and third-order correlation function of the cavity field. Furthermore, we show the effects of gravity on conventional single-photon blockade, nonstandard types of single-photon blockade, two-photon blockade, and photon-induced tunneling in the quadratically coupled optomechanical system. Finally, we give conclusions of our work.

## Results

### Physical model and solution

**Figure 1:**
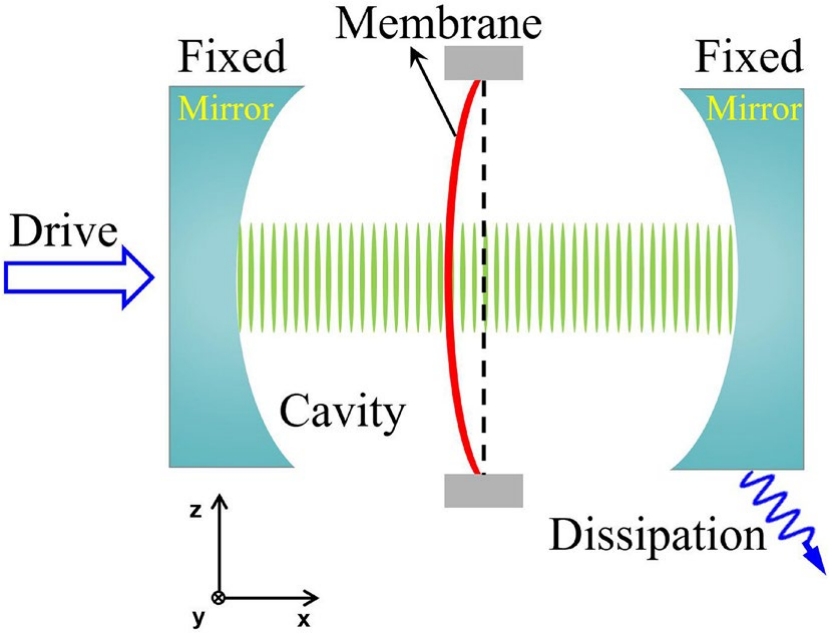
Schematic diagram of the quadratically coupled optomechanical system with dissipation.

**Figure 2:**
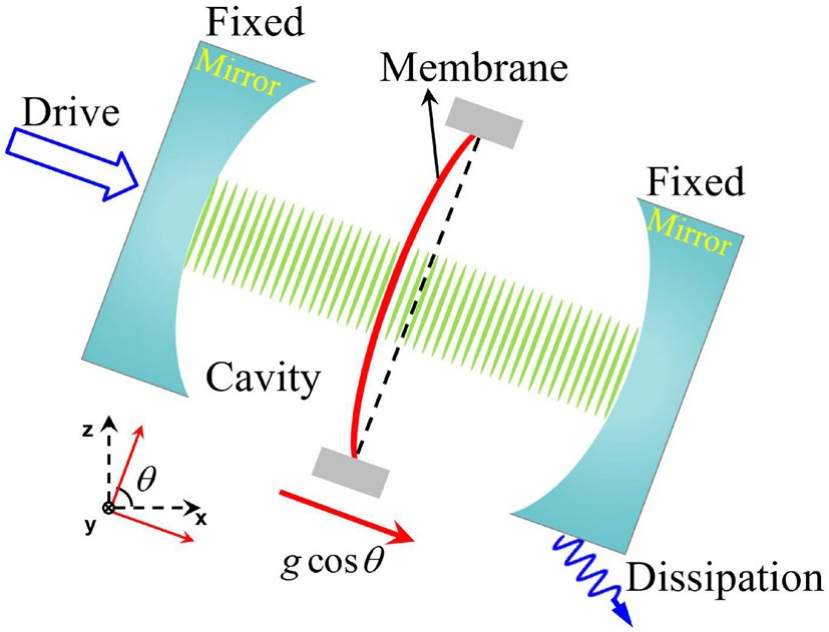
Schematic diagram of the quadratically coupled optomechanical system with dissipation after clockwise rotation $\pi /2-\theta$ around the *y*-axis.

As schematically shown in Fig. 1, the quadratic optomechanical coupling between the photons and the membrane in the cavity occurs only in the horizontal direction. Thus, the quadratic optomechanical coupling is independent of the external gravity when the quadratically coupled optomechanical system stays horizontal. Now, we rotate the whole quadratically coupled optomechanical system around the *y*-axis, as schematically shown in Fig. 2. Then, the Newtonian gravity will be able to modulate quadratic optomechanical coupling between the photons and the membrane in the cavity. In this section, we will introduce the effective Hamiltonian of the quadratically coupled optomechanical system in the Newtonian gravitational field and give the corresponding eigenvalues, eigenstates, and steady-state solutions.

#### System hamiltonian

The quadratically coupled optomechanical system is composed of a ”membrane-in-the-middle” configuration and Fabry-Pérot optical cavity. As schematically shown in Fig. 1, the mechanical displacement of the membrane quadratically couples to the photons in the cavity. Here, we assume the quadratically coupled optomechanical system is driven by an external field. The Hamiltonian of the system is written as^5,56^

1 $$\begin{aligned} H_{S}= \omega _{a}a^{\dag }a+\omega _{b}b^{\dag }b+\lambda a^{\dag }a\left( b^{\dag }+b\right) ^{2}+\Omega \left( a^{\dag }e^{-i\omega _{L}t}+ae^{i\omega _{L}t}\right) , \end{aligned}$$

where $\omega _{a}$ and $\omega _{b}$ are the eigen frequencies of cavity mode *a* and mechanical mode *b*, respectively. The third term in $H_{S}$ describes the quadratic optomechanical coupling with strength $\lambda$ between the cavity field and the mechanical motion of the membrane. The last term represents the coupling between the cavity and the driving field with laser amplitude $\Omega$ and frequency $\omega _{L}$.

As schematically shown in Fig. 2, in order to introduce a coupling to a gravitational potential in the Hamiltonian, we add a term of the form

2 $$\begin{aligned} H_{G}=xmg\cos \theta . \end{aligned}$$

where *m* is the mass of the mechanical membrane, *g* is the gravitational acceleration, $\theta$ is an angle from the horizontal axis, and *x* is the position operator acting on the mechanical oscillator that we include in order to describe inclined systems^59,63^. The position operator $x=(b+b^{\dag })/\sqrt{2m\omega _b}$ in our situation^59,61^. With the addition of Newtonian gravity, the Hamiltonian of the system is

3 $$\begin{aligned} H= &  H_{S}+H_{G}\nonumber \\= &  \omega _{a}a^{\dag }a+\omega _{b}b^{\dag }b+\lambda a^{\dag }a\left( b^{\dag }+b\right) ^{2}+g_{0}\left( b^{\dag }+b\right) +\Omega \left( a^{\dag }e^{-i\omega _{L}t}+ae^{i\omega _{L}t}\right) , \end{aligned}$$

where $g_{0}=g\cos \theta \sqrt{m/2\omega _{b}}$ is the renormalized gravity coupling parameter. The sine component of the gravity remains orthogonal to the direction of the quadratic optomechanical coupling. Therefore, the sine component of gravity is not correlated with the quadratic optomechanical coupling. The sine component of gravity will have no effect on the dynamics of the system in the direction of the quadratic optomechanical coupling. In the rotating picture at the driving frequency $\omega _{L}$, the Hamiltonian of the system can be rewritten as

4 $$\begin{aligned} H= \Delta _{a}a^{\dag }a+\omega _{b}b^{\dag }b+\lambda a^{\dag }a\left( b^{\dag }+b\right) ^{2}+g_{0}\left( b^{\dag }+b\right) +\Omega \left( a^{\dag }+a\right) , \end{aligned}$$

with the detuning $\Delta _{a}= \omega _{a}-\omega _{L}$ between the cavity mode and the driving field.

#### The eigenvalues and eigenstates of the system

We assume that the $|n\rangle _{a}$ and $|m\rangle _{b}$ are the harmonic-oscillator number states of the cavity and the membrane, respectively. Since the external driving laser field is very weak, we can study the eigensystem of the first four terms

5 $$\begin{aligned} H_{OMG}= \Delta _{a}a^{\dag }a+\omega _{b}b^{\dag }b+\lambda a^{\dag }a\left( b^{\dag }+b\right) ^{2}+g_{0}\left( b^{\dag }+b\right) . \end{aligned}$$

The optical field excitation number is conservative in $H_{OMG}$. The membrane has the form of quadratically coupling and gravity driving. Thus, in the first step of diagonalization, we use the squeezing operator

6 $$\begin{aligned} S(\xi )=\exp \left[ \frac{\xi }{2}(b^{2}-b^{\dag 2})\right] , \end{aligned}$$

where $\xi$ is the squeezing parameter. We assume that the squeezing parameter $\xi$ is a real number. The effect of this squeezing unitary transformation on the operators *b* and $b^{\dag }$ will obtain the following operators

7 $$\begin{aligned} b'= &  S(-\xi )bS(\xi )=b\cosh \xi -b^{\dag }\sinh \xi ,\end{aligned}$$

8 $$\begin{aligned} b'^{\dag }= &  S(-\xi )b^{\dag }S(\xi )=b^{\dag }\cosh \xi -b\sinh \xi . \end{aligned}$$

Then, applying the squeezing unitary transformation $S(\xi )$ to Hamiltonian $H_{OMG}$, the Hamiltonian of $H_{OMG}$ becomes

9 $$\begin{aligned} H_{OMG}'= &  S(-\xi )H_{OMG}S(\xi )\nonumber \\= &  \Delta _{a}a^{\dag }a+\omega _{b}b'^{\dag }b'+\lambda a^{\dag }a(b'^{\dag }+b')^{2}+g_{0}\left( b'^{\dag }+b'\right) . \end{aligned}$$

Substituting the $b'$ and $b'^{\dag }$ into the $H_{OMG}'$, the Hamiltonian $H_{OMG}'$ will be rewritten as

10 $$\begin{aligned} H_{OMG}'=\Delta _{a}a^{\dag }a+\omega _{b}^{\left( n\right) }b^{\dag }b+\delta _{b}^{\left( n\right) }+g_{0}^{\left( n\right) }\left( b^{\dag }+b\right) . \end{aligned}$$

The *n*-photon coupled membrane’s resonant frequency $\omega _{b}^{\left( n\right) }$, frequency shift $\delta _{b}^{\left( n\right) }$, gravity driving $g_{0}^{\left( n\right) }$ and squeezing factors are respectively

11 $$\begin{aligned} \omega _{b}^{\left( n\right) }= &  \omega _{b}\sqrt{1+\frac{4\lambda n}{\omega _{b}}},\end{aligned}$$

12 $$\begin{aligned} \delta _{b}^{\left( n\right) }= &  \frac{1}{2}\left( \omega _{b}^{\left( n\right) }-\omega _{b}\right) ,\end{aligned}$$

13 $$\begin{aligned} g_{0}^{\left( n\right) }= &  g_{0}\exp \left[ -\frac{1}{4}\ln \left( 1+\frac{4\lambda n}{\omega _{b}}\right) \right] ,\end{aligned}$$

14 $$\begin{aligned} \xi= &  \frac{1}{4}\ln \left( 1+\frac{4\lambda n}{\omega _{b}}\right) \end{aligned}$$

One can easily find that $H_{OMG}'$ is not diagonal in terms of the operators of the membrane due to the presence of gravity driving term. Thus, we can select the displacement operator to diagonalize the Hamiltonian $H_{OMG}'$. The definition of displacement operator is

15 $$\begin{aligned} D(\alpha )=\exp \left[ \alpha (b^{\dag }-b)\right] , \end{aligned}$$

where $\alpha$ is defined as

16 $$\begin{aligned} \alpha =-\frac{g_{0}^{\left( n\right) }}{\omega _{b}^{\left( n\right) }}. \end{aligned}$$

According to the Baker–Campbell–Hausdorff formula, the action of this displacement unitary transformation on the operators *b* and $b^{\dag }$ will give the following results:

17 $$\begin{aligned} b''= &  D(-\alpha )bD(\alpha )=b+\alpha ,\end{aligned}$$

18 $$\begin{aligned} b''^{\dag }= &  D(-\alpha )b^{\dag }D(\alpha )=b^{\dag }+\alpha . \end{aligned}$$

Transforming the Hamiltonian $H_{OMG}'$ under the unitary operator $D\left( \alpha \right)$, the Hamiltonian $H_{OMG}'$ becomes

19 $$\begin{aligned} H_{OMG}''= &  D(-\alpha )H_{OMG}'D(\alpha )\nonumber \\= &  \Delta _{a}a^{\dag }a+\omega _{b}^{\left( n\right) }b''^{\dag }b''+\delta _{b}^{\left( n\right) }+g_{0}^{\left( n\right) }\left( b''^{\dag }+b''\right) . \end{aligned}$$

After substituting the $b''$ and $b''^{\dag }$ into the $H_{OMG}''$, the Hamiltonian $H_{OMG}''$ will be rewritten as

20 $$\begin{aligned} H_{OMG}''=\Delta _{a}a^{\dag }a+\omega _{b}^{\left( n\right) }b^{\dag }b+\delta _{b}^{\left( n\right) }-\frac{\left( g_{0}^{\left( n\right) }\right) ^{2}}{\omega _{b}^{\left( n\right) }}. \end{aligned}$$

According to the diagonalized Hamiltonian $H_{OMG}''$, the eigenvalues and eigenstates of $H_{OMG}''$ are

21 $$\begin{aligned} E_{n,m}= &  \Delta _{a}n+\omega _{b}^{\left( n\right) }m+\delta _{b}^{\left( n\right) }-\frac{\left( g_{0}^{\left( n\right) }\right) ^{2}}{\omega _{b}^{\left( n\right) }},\end{aligned}$$

22 $$\begin{aligned} |n,m\rangle= &  |n\rangle _{a}\otimes |m\rangle _{b}, \end{aligned}$$

Then, the eigensystem of the Hamiltonian $H_{OMG}''$ can be expressed as

23 $$\begin{aligned} H_{OMG}''|n\rangle _{a}|m\rangle _{b}= &  E_{n,m}|n\rangle _{a}|m\rangle _{b},\nonumber \\ H_{OMG}S(\xi )D(\alpha )|n\rangle _{a}|m\rangle _{b}= &  E_{n,m}S(\xi )D(\alpha )|n\rangle _{a}|m\rangle _{b},\nonumber \\ H_{OMG}|n\rangle _{a}|\tilde{m}(n)\rangle _{b}= &  E_{n,m}|n\rangle _{a}|\tilde{m}(n)\rangle _{b}. \end{aligned}$$

The *n*-photon squeezed displaced number state $|\tilde{m}(n)\rangle _{b}$ in the above equation is defined as

24 $$\begin{aligned} |\tilde{m}(n)\rangle _{b}=S(\xi )D(\alpha )|m\rangle _{b}. \end{aligned}$$

The wave function corresponding to the system described by the Hamiltonian $H_{OMG}$ can be expanded through the eigenstates of the Hamiltonian $H_{OMG}$. Thus, a general state of the system can be expressed as

25 $$\begin{aligned} |\varphi \left( t\right) \rangle =\sum _{n=0}^{\infty }\sum _{m=0}^{\infty }C_{n,m}\left( t\right) |n\rangle _{a}|\tilde{m}(n)\rangle _{b}. \end{aligned}$$

#### Approximate analytical solutions for steady-state

In this section, we analytically calculate the probability amplitude of the steady state when the weak-driving term in the Hamiltonian *H* is treated as a perturbation. In our situation, there will be a dissipation of the cavity photons and neglect the membrane’s dissipation^5^ when the quadratically coupled optomechanical system is driven by an external laser and Newtonian gravity. In our numerical results, the mechanical dissipation is taken into account. For simplicity, the optical losses can be phenomenologically added to Hamiltonian *H* to describe the dissipation of the cavity photons. And, we assume that the temperature of membrane is zero. Thus, the effective non-Hermitian Hamiltonian containing the optical losses can be expressed as

26 $$\begin{aligned} H_{eff}=H-i\frac{\gamma _{a}}{2}a^{\dag }a. \end{aligned}$$

Under the weak-driving condition $\Omega \ll \gamma _{a}$, the number of photons excited in the cavity is small. So we can work within the few excitation number subspace spanned by the basis states $|0\rangle$, $|1\rangle$, $|2\rangle$, and $|3\rangle$ of the cavity photons number states. The general state $|\varphi \rangle$ of the system can be simplified as

27 $$\begin{aligned} |\varphi \left( t\right) \rangle =\sum _{n=0}^{3}\sum _{m=0}^{\infty }C_{n,m}\left( t\right) |n\rangle _{a}|\tilde{m}(n)\rangle _{b}, \end{aligned}$$

where $C_{n,m}\left( t\right)$ is probability amplitudes of pure state $|\varphi \left( t\right) \rangle$. According to the effective Hamiltonian $H_{eff}$, simplified wave function $|\varphi \rangle$, and the Schrödinger equation, the equations of motion for the probability amplitudes $C_{n,m}\left( t\right)$ are

28 $$\begin{aligned} \dot{C}_{0,m}(t)= & -iE_{0,m}C_{0,m}(t)-i\Omega \sum ^{\infty }_{m'=0}{}_{b}\langle \tilde{m}(0)|\tilde{m'}(1)\rangle _{b}C_{1,m'}(t),\end{aligned}$$

29 $$\begin{aligned} \dot{C}_{1,m}(t)= &  -(\frac{\gamma _{a}}{2}+iE_{1,m})C_{1,m}(t)-i\Omega \sum ^{\infty }_{m'=0}{}_{b}\langle \tilde{m}(1)|\tilde{m'}(0)\rangle _{b}C_{0,m'}(t)\nonumber \\ &  -i\sqrt{2}\Omega \sum ^{\infty }_{m'=0}{}_{b}\langle \tilde{m}(1)|\tilde{m'}(2)\rangle _{b}C_{2,m'}(t),\end{aligned}$$

30 $$\begin{aligned} \dot{C}_{2,m}(t)= &  -(\gamma _{a}+iE_{2,m})C_{2,m}(t)-i\sqrt{2}\Omega \sum ^{\infty }_{m'=0}{}_{b}\langle \tilde{m}(2)|\tilde{m'}(1)\rangle _{b}C_{1,m'}(t)\nonumber \\ &  -i\sqrt{3}\Omega \sum ^{\infty }_{m'=0}{}_{b}\langle \tilde{m}(2)|\tilde{m'}(3)\rangle _{b}C_{3,m'}(t),\end{aligned}$$

31 $$\begin{aligned} \dot{C}_{3,m}(t)= &  -(\frac{3\gamma _{a}}{2}+iE_{3,m})C_{3,m}(t)-i\sqrt{3}\Omega \sum ^{\infty }_{m'=0}{}_{b}\langle \tilde{m}(3)|\tilde{m'}(2)\rangle _{b}C_{2,m'}(t). \end{aligned}$$

The weak external driving, which driving rate is much less than the cavity dissipation rate, (i.e. $\Omega \ll \gamma _{a}$), excites few photon-states in cavity. Thus, the probability amplitude of few-photon states can be approximately solved when the higher-order terms in the zero-, one-, and two-photon probability amplitudes are discarded^5^, i.e., dropping the second, third, and third terms in $\dot{C}_{0,m}(t)$, $\dot{C}_{1,m}(t)$, and $\dot{C}_{2,m}(t)$, respectively. Then, the long-time approximately solution for the probability amplitude of few-photon states can be expressed as

32 $$\begin{aligned} C_{0,m}(t)= &  C_{0,m}(0)e^{-iE_{0,m}t},\end{aligned}$$

33 $$\begin{aligned} C_{1,m}(t)= &  -\Omega \sum ^{\infty }_{m'=0}\frac{_{b}\langle \tilde{m}(1)|\tilde{m'}(0)\rangle _{b}C_{0,m'}(0)e^{-iE_{0,m'}t}}{E_{1,m}-E_{0,m'}-i\frac{\gamma _{a}}{2}},\end{aligned}$$

34 $$\begin{aligned} C_{2,m}(t)= &  \sqrt{2}\Omega ^{2}\sum ^{\infty }_{m',m''=0}\frac{_{b}\langle \tilde{m}(2)|\tilde{m'}(1)\rangle _{b}}{E_{2,m}-E_{0,m''}-i\gamma _{a}}\times \frac{_{b}\langle \tilde{m'}(1)|\tilde{m''}(0)\rangle _{b}C_{0,m''}(0)e^{-iE_{0,m''}t}}{E_{1,m'}-E_{0,m''}-i\frac{\gamma _{a}}{2}},\end{aligned}$$

35 $$\begin{aligned} C_{3,m}(t)= &  -\sqrt{6}\Omega ^{3}\sum ^{\infty }_{m',m'',m'''=0}\frac{_{b}\langle \tilde{m}(3)|\tilde{m'}(2)\rangle _{b}}{E_{3,m}-E_{0,m'''}-i\frac{3\gamma _{a}}{2}}\times \frac{_{b}\langle \tilde{m'}(2)|\tilde{m''}(1)\rangle _{b}}{E_{2,m'}-E_{0,m'''}-i\gamma _{a}}\nonumber \\ &  \times \frac{_{b}\langle \tilde{m''}(1)|\tilde{m'''}(0)\rangle _{b}C_{0,m'''}(0)e^{-iE_{0,m'''}t}}{E_{1,m''}-E_{0,m'''}-i\frac{\gamma _{a}}{2}}, \end{aligned}$$

where $C_{1,m}(0)=C_{2,m}(0)=C_{3,m}(0)=0$ is the initial situation for an initial empty cavity and $C_{0,m}(0)$, $C_{0,m'}(0)$, $C_{0,m''}(0)$, and $C_{0,m'''}(0)$ are determined by the initial state of the membrane. For simplicity, we assume the membrane is in vacuum states $\left| 0\right\rangle _{b}$, initially. Then, the probability amplitudes take the form $C_{0,m}(0)=\delta _{0,m}$. So far, the probability amplitude of the system wave function in few-photon subspace can be approximately obtained:

36 $$\begin{aligned} C_{0,m}(t)\approx &  e^{-iE_{0,0}t},\end{aligned}$$

37 $$\begin{aligned} C_{1,m}(t)\approx &  -\Omega \frac{_{b}\langle \tilde{m}(1)|\tilde{0}(0)\rangle _{b} e^{-iE_{0,0}t}}{E_{1,m}-E_{0,0}-i\frac{\gamma _{a}}{2}},\end{aligned}$$

38 $$\begin{aligned} C_{2,m}(t)\approx &  \sqrt{2}\Omega ^{2}\sum ^{\infty }_{m'=0}\frac{_{b}\langle \tilde{m}(2)|\tilde{m'}(1)\rangle _{b} }{E_{2,m}-E_{0,0}-i\gamma _{a}}\times \frac{_{b}\langle \tilde{m'}(1)|\tilde{0}(0)\rangle _{b} e^{-iE_{0,0}t}}{E_{1,m'}-E_{0,0}-i\frac{\gamma _{a}}{2}},\end{aligned}$$

39 $$\begin{aligned} C_{3,m}(t)\approx &  -\sqrt{6}\Omega ^{3}\sum ^{\infty }_{m',m''=0}\frac{_{b}\langle \tilde{m}(3)|\tilde{m'}(2)\rangle _{b}}{E_{3,m}-E_{0,0}-i\frac{3\gamma _{a}}{2}}\times \frac{_{b}\langle \tilde{m'}(2)|\tilde{m''}(1)\rangle _{b}}{E_{2,m'}-E_{0,0}-i\gamma _{a}}\nonumber \\ &  \times \frac{_{b}\langle \tilde{m''}(1)|\tilde{0}(0)\rangle _{b}e^{-iE_{0,0}t}}{E_{1,m''}-E_{0,0}-i\frac{\gamma _{a}}{2}}. \end{aligned}$$

The transition rates $_{b}\langle \tilde{m}(n)|\tilde{m'}(n')\rangle _{b}$ is actually an inner product of two squeezed displaced number states. The inner product of two squeezed displaced number states had been calculated by J. P. Dahl^64^. Finally, we can obtain an long-time approximate analytical result for the few-photon state $\left| \varphi \left( t\right) \right\rangle$.

### Results for single-photon blockade and two-photon blockade

#### Conventional single-photon blockade with gravity driving

Single-photon blockade refers to the presence of one photon in a driven nonlinear optical system, which will prevent the entry of a second photon into the cavity. Therefore, the probability of finding two photons in an optical system will be suppressed due to the nonlinearity of the system’s energy levels. This will cause that the correlation between the photons inside the optical system and the external photons is absent. Accordingly, the experimental condition for producing single-photon blockade is the zero-time-delay second-order correlation function $g^{2}\left( 0\right) <1$. Conventional photon blockade can also be recognized from the deviations of the photon distribution $P_{n}=\langle n |\rho |n\rangle$ to the standard Poisson distribution $\mathscr {P}_{n}=\langle n\rangle ^{n}\exp \left( -\langle n\rangle \right) /n!$ with the same mean photon number. The deviations are defined as

40 $$\begin{aligned} \beta _{n}=\frac{P_{n}-\mathscr {P}_{n}}{\mathscr {P}_{n}}. \end{aligned}$$

When the system is in the state $\left| \varphi \left( t\right) \right\rangle$, the zero-time-delay second- and third-order correlation function of the cavity field can be expressed as

41 $$\begin{aligned} g^{(2)}(0)= &  \frac{\langle a^{\dag 2}a^{2}\rangle }{\langle a^{\dag }a\rangle ^{2}}=\frac{2P_{2}+6P_{3}}{(P_{1}+2P_{2}+3P_{3})^{2}},\end{aligned}$$

42 $$\begin{aligned} g^{(3)}(0)= &  \frac{\langle a^{\dag 3}a^{3}\rangle }{\langle a^{\dag }a\rangle ^{3}}=\frac{6P_{3}}{(P_{1}+2P_{2}+3P_{3})^{3}}, \end{aligned}$$

where $P_{i}=\sum _{m=0}^{\infty }\left| C_{i,m}(t)\right| ^{2}$ is the probability of finding *i* photons in the cavity. Combining $C_{0,m}(t)$, $C_{1,m}(t)$, $C_{2,m}(t)$, and $C_{3,m}(t)$, the photon number state probability amplitude $P_{i}$ ($i=0,1,2,3$), $g^{(2)}(0)$, and $g^{(3)}(0)$ can be calculated in the few-photon subspace.

**Figure 3:**
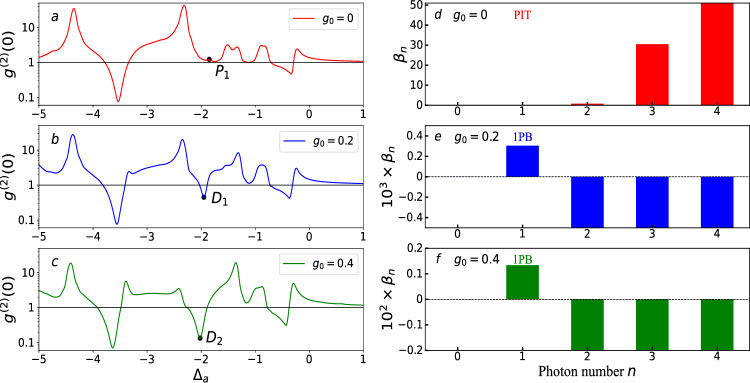
In (**a**–**c**), the second-order correlation function $g^{\left( 2\right) }\left( 0\right)$ of the optical mode versus the driving detuning $\Delta _{a}$ for various values of the gravity parameter $g_{0}$: (**a**) $g_{0}=0$, (**b**) $g_{0}=0.2$, (c) $g_{0}=0.4$. Other parameters are $\lambda =0.4$, $\Omega =0.01$, and $\gamma _{a}=0.1$. All variables take values in units of $\omega _{b}$. In (**d**–**f**), the deviation criterion of the conventional photon blockade for different gravity parameters $g_{0}$ and driving detuning $\Delta _{a}$: (**d**) $g_{0}=0$, $\Delta _{a}=-1.85$, (**e**) $g_{0}=0.2$, $\Delta _{a}=-1.95$, (**f**) $g_{0}=0.4$, $\Delta _{a}=-2.02$. Other parameters are $\lambda =0.4$, $\Omega =0.01$, and $\gamma _{a}=0.1$. All variables take values in units of $\omega _{b}$.

In Fig. 3a–c, we plot the second-order correlation function $g^{\left( 2\right) }\left( 0\right)$ of the optical mode as a function of driving detuning $\Delta _{a}$, when the gravity parameter $g_{0}$ takes various values. Figure 3a shows that there are two dips in the region where the function $g^{\left( 2\right) }\left( 0\right)$ is less than 1. And, there are also some peaks in the region of the function $g^{\left( 2\right) }\left( 0\right)$ greater than 1. This suggests that the photon statistics switches between super-Poisson and sub-Poisson distributions with the change of $\Delta _{c}$. In particular, the dips and peaks in these curves correspond to the single-photon blockade and no photon blockade cases, respectively. It is worth noting that we have not yet considered the effect of gravity on the photon statistics.

In Fig. 3b, c, we assume that the gravity parameter $g_{0}$ is equal to 0.2 and 0.4, respectively. Comparing with Fig. 3a, we first find that gravity can induce a new dip in the second-order correlation function of the optical mode. Second, comparing Fig. 3b, c, it is easy to see that an enhancement of the gravity parameter leads to a deepening of the new dip. However, gravity doesn’t have an effect on the previous dips in Fig. 3a. Therefore, we believe that the gravity can induce photon blockade and change the degree of photon blockade without changing the original photon statistics in the system. Gravity behaves like a photon blockade valve.

In Fig. 3d–f, we show the deviations $\beta _{n}$ of the photon distribution $P_{n}$ to the standard Poisson distribution $\mathscr {P}_{n}$ at point $P_{1}$, $D_{1}$, and $D_{2}$ in Fig. 3a–c. In Fig. 3d, the deviations $\beta _{n}$ are always greater than zero, regardless of the number of photons *n*. This suggests that the intracavity photons are in a super-Poissonian distribution at position $P_{1}$. Figure 3e and f show that the deviations $\beta _{1}$ are greater than zero. However, the deviations $\beta _{2}$, $\beta _{3}$, and $\beta _{4}$ are both smaller than zero. This indicates that the single photon probability $P_{1}$ is enhanced while $P_{n>1}$ are suppressed at points $D_{1}$ and $D_{2}$. This suggests that the intracavity photons are in a sub-Poissonian distribution at point $D_{1}$ and $D_{2}$.

**Figure 4:**
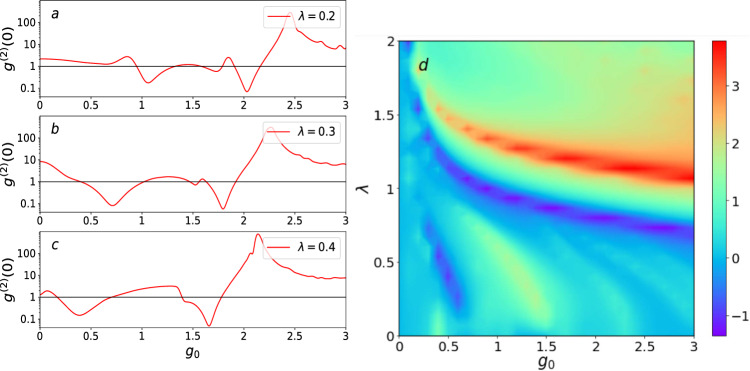
In (**a**–**c**), the second-order correlation function $g^{\left( 2\right) }\left( 0\right)$ of the optical mode versus the gravity parameter $g_{0}$ for various values of the optomechanical coupling parameter $\lambda$: (**a**) $\lambda =0.2$, (**b**) $\lambda =0.3$, (**c**) $\lambda =0.4$. Other parameters are $\Delta _{a}=-2$, $\Omega =0.01$, and $\gamma _{a}=0.1$. All variables take values in units of $\omega _{b}$. In (**d**), the plot of $g^{\left( 2\right) }\left( 0\right)$ as a function of the optomechanical coupling strength $\lambda$ and gravity parameter $g_{0}$ under the driving conditions $\Delta _{a}=-2$. Other parameters are $\Omega =0.01$ and $\gamma _{a}=0.1$. All variables take values in units of $\omega _{b}$.

To clarify the inherent relationship between the gravity coupling and the quadratic optomechanical coupling for observing the photon blockade, we illustrate the second-order function $g^{(2)}(0)$ as a function of $g_{0}$ and $\lambda$ under the driving detuning condition $\Delta _{a}=-2$ in Fig. 4. In Fig. 4a–c, we find that the dips of $g^{(2)}(0)$ move left with increasing $\lambda$. At the same time, the value of the dips of $g^{(2)}(0)$ has not changed much. This means that the increase of the gravity coupling strength $g_{0}$ can keep the strength of the photon blockade unchanged when decreasing the quadratic optomechanical coupling $\lambda$. The dark blue area in the middle region of Fig. 4d indicates that the photon distribution is in a sub-Poissonian distribution. Moreover, it can be more clearly seen that the gravity coupling is in a complementary relationship with the quadratic optomechanical coupling for observing the photon blockade. The gravity coupling can be considered as a compensation when the quadratic optomechanical coupling is weak. However, it is important to note that the gravity coupling cannot completely replace quadratic optomechanical coupling. The red area in the upper region of Fig. 4d indicates that the photon distribution is in a Poissonian distribution. In this parameter region, photon blockade does not occur regardless of how the gravity coupling strength and the quadratic optomechanical coupling strength are varied. This suggests that when both gravity coupling strength and the quadratic optomechanical coupling strength are large at the same time, the system does not exhibit an anti-bunching effect.

**Table 1:** Criterion for three types of the single-photon blockade.

Single-photon blockade	Criterion
Type 1	$g^{3}\left( 0\right)<g^{2}\left( 0\right) <1$
Type 2	$g^{2}\left( 0\right)<1<g^{3}\left( 0\right)$
Type 3	$g^{2}\left( 0\right)<g^{3}\left( 0\right) <1$

#### Nonstandard types of single-photon blockade with gravity driving

Combined zero-time-delay two-order correlation function $g^{2}\left( 0\right)$ and three-order correlation function $g^{3}\left( 0\right)$, the single-photon blockade can be subdivided into three types^65^. The corresponding criteria for different types of single-photon blockade are shown in Table 1.

The single-photon blockade of type 1 is described as a good single-photon source. Because the second-order function $g^{\left( 2\right) }\left( 0\right)$ is less than 1. This indicates that the two-photon correlation is suppressed. It is important that, at the same time, the correlation between subsequent photons is smaller when the third-order function $g^{\left( 3\right) }\left( 0\right)$ is less than the second-order function $g^{\left( 2\right) }\left( 0\right)$. Thus, the single-photon blockade of type 2 and type 3 can’t be called good single-photon sources. They are referred to as nonstandard photon blockade.

**Figure 5:**
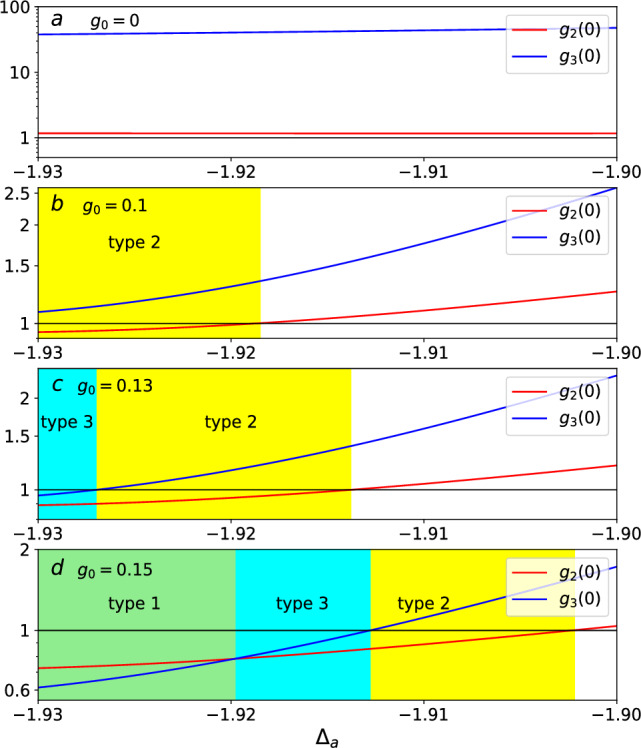
In (**a**–**d**), the second-order correlation function $g^{\left( 2\right) }\left( 0\right)$ of the optical mode versus the driving detuning $\Delta _{a}$ for various values of the gravity parameter $g_{0}$: (**a**) $g_{0}=0$, (**b**) $g_{0}=0.1$, (**c**) $g_{0}=0.13$, (**d**) $g_{0}=0.15$. Other parameters are $\lambda =0.4$, $\Omega =0.01$, and $\gamma _{a}=0.1$. All variables take values in units of $\omega _{b}$.

It’s worth noting that the nonstandard single-photon blockade of type 2 is also called superbunching effect^66,67^. The superbunching effect is that the probability of measuring two photons in the cavity at the same time is suppressed, while the probability of obtaining three photons in the cavity is enhanced. The criterion of type 2 indicates that the second-order function $g^{\left( 2\right) }\left( 0\right)$ is less than 1 and the third-order function $g^{\left( 3\right) }\left( 0\right)$ is greater than 1. This means that the two-photon correlation is suppressed and, simultaneously, the three-photon correlation is enhanced via the high-order transition processes.

To clarify the effect of gravity coupling for observing the nonstandard single-photon blockade, we illustrate, in Fig. 5, the second-order correlation function $g^{\left( 2\right) }\left( 0\right)$ and third-order correlation function $g^{\left( 3\right) }\left( 0\right)$ as a function of $\Delta _{a}$ under the gravity coupling conditions: (a) $g_{0}=0$, (b) $g_{0}=0.1$, (c) $g_{0}=0.13$, and (d) $g_{0}=0.15$. We can see two features from Fig. 5:

(i) The curves $g^{\left( 2\right) }\left( 0\right) =1$ provide a boundary for different photon distributions: sub-Poissonian distributions $(g^{\left( 2\right) }\left( 0\right) <1)$ and super-Poissonian distributions $(g^{\left( 2\right) }\left( 0\right) >1)$. In Fig. 5a, the correlation functions $g^{\left( 2\right) }\left( 0\right)$ and $g^{\left( 3\right) }\left( 0\right)$ are both greater than 1 when the gravity coupling is absent. In Fig. 5b–d, the nonstandard single-photon blockade phenomenon appears when gravity coupling is present. This means that the nonstandard single-photon blockade grows when increasing the gravity coupling strength.
(ii) In the region $0.1\le g_{0}\le 0.15$, the types of the nonstandard single-photon blockade gradually increase with increasing the gravity coupling strength $g_{0}$. It is seen that the good single-photon sources of type 1 occurs when the gravity coupling strength $g_{0}=0.15$. We can explain this from the fact that both the gravity coupling and quadratic optomechanical coupling are beneficial to observing the photon blockade. Thus, the single-photon blockade of type 1 grows when increasing the gravity coupling strength $g_{0}$ for the same quadratic optomechanical coupling strength $\lambda$.

Based on the above analysis, we think that the gravity coupling is beneficial to the preparation of good single-photon sources in the quadratically coupled optomechanical system when the quadratic optomechanical coupling strength is weak.

#### Two-photon blockade and tunneling with gravity driving

**Figure 6:**
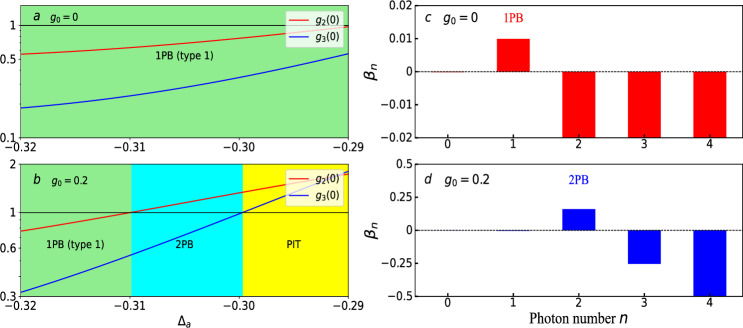
In (**a**–**b**), the second-order correlation function $g^{\left( 2\right) }\left( 0\right)$ of the optical mode versus the driving detuning $\Delta _{a}$ for various values of the gravity parameter $g_{0}$: (**a**) $g_{0}=0$, (**b**) $g_{0}=0.2$. Other parameters are $\lambda =0.4$, $\Omega =0.01$, and $\gamma _{a}=0.1$. All variables take values in units of $\omega _{b}$. In (**c**–**d**), the deviation criterion of the conventional photon blockade for different gravity parameters $g_{0}$: (**c**) $g_{0}=0$, (**d**) $g_{0}=0.2$. Other parameters are $\lambda =0.4$, $\Omega =0.01$, $\Delta _{a}=-0.305$,and $\gamma _{a}=0.1$. All variables take values in units of $\omega _{b}$.

In this section, we investigate the effects of gravity coupling on two-photon blockade under the driving detuning condition $-0.32\le \Delta _{a}\le -0.29$. In the case of two-photon blockade, the probability of observing two photons at the same time is enhanced and, simultaneously, the probability of measuring three photons has to be suppressed in the cavity, i.e., $g^{\left( 2\right) }\left( 0\right) >1$ and $g^{\left( 3\right) }\left( 0\right) <1$. The generation of two photons at the same instance of time and the preventing subsequent photons from entering the cavity has the potential to generate entangled photon pairs. As shown in Fig. 6a, the photons in the cavity are in nature the quantum light, i.e., sub-Poissonian distributions, with the second and third-order correlations function $g^{\left( 2\right) }\left( 0\right) , g^{\left( 3\right) }\left( 0\right) <1$ when the gravity coupling is absent. This indicates that the strong two-photon correlation is absent $(g^{\left( 2\right) }\left( 0\right) <1)$. Thus, it is noteworthy that these photons do not exist in the form of photon pairs in the case without gravity coupling in the cavity.

However, the two-photon blockade can happen in the gravity coupled quadratically optomechanical system. From Fig. 6b, we observe that the two-photon blockade can also be obtained when the gravity coupling is considered, as displayed by the cyan area. The reason for the behavior that happened here is that the gravity coupled membrane increases the nonlinearity of the quadratically optomechanical system, making it possible to realize the two-photon transition between the membrane and cavity. These results can also be confirmed by calculating the deviations $\beta _{n}$ between the photon-number distribution $P_{n}$ and the Poisson distribution $\mathscr {P}_{n}$ when driving detuning $\Delta _{a}=-0.305$, as shown in Fig. 6c and d. We can find that the two-photon probability $P_{2}$, three-photon probability $P_{3}$, and four-photon probability $P_{4}$ at the same time are suppressed and, simultaneously, the single-photon probability $P_{1}$ is enhanced in Fig. 6c. However, the three-photon probability $P_{3}$ and four-photon probability $P_{4}$ at the same time are suppressed and, simultaneously, the two-photon probability $P_{2}$ is enhanced in Fig. 6d.

Moreover, we observe that the photon-induced tunneling effect can also occur when the gravity coupled quadratically optomechanical system has different driving frequencies. It is noteworthy that the yellow area of Figs. 6b corresponds to the two-photon tunneling, i.e., the simultaneous arrival of two photons in the cavity is enhanced compared to single-photon arrivals. The two-photon tunneling is usually characterized by the super-Poissonian photon-number statistics, where $1<g^{\left( 3\right) }\left( 0\right) <g^{\left( 2\right) }\left( 0\right)$. Thus, the photons in the cavity are in nature the classical light when the system parameters are within the yellow region in Fig. 6b. The green area in Fig. 6b shows that the photons in the cavity are quantum light. The cyan area in Fig. 6b can be regarded as the transition phase between quantum and classical with increasing driving detuning $\Delta _{a}$.

Based on the above findings, we believe that the gravity coupled quadratically optomechanical system can realize not only two-photon blockade, but also light transformation between quantum and classical.

## Discussion

In conclusion, we have presented a gravity coupled quadratic optomechanical system by rotating the entire quadratically coupled optomechanical system around the *y*-axis. With the help of squeezing and displacement unitary transformation, we have obtained the eigenvalues and eigenstates of the gravity coupled quadratically optomechanical system. Then, we also have acquired the approximate analytical expression of the steady-states for the whole system by treating the external optical driving term as a perturbation. Eventually, we numerically calculated the second-order, third-order correlation function, and deviations of the photon distribution for the cavity photons when the cavity dissipation is considered.

With the assistance of cavity photons correlation function and deviations of the photon distribution, we analyze the effect of gravitational coupling on conventional single-photon blockade, nonstandard single-photon blockade, two-photon blockade, and photon-induced tunneling in the gravity coupled quadratically optomechanical system. We found that the additional photon blockade can be induced by the gravity coupled membrane. Also, the accession of gravity does not affect the previous photon blockade, which is induced by the quadratic optomechanical coupling. In particular, both the gravity coupling and quadratic optomechanical coupling are beneficial for observing the photon blockade. The gravity coupling can be considered as a compensation when the quadratic optomechanical coupling is weak.

Finally, the nonstandard photon blockade can also be observed in a weak driving regime when the gravity coupling is considered. Moreover, in this regime, the transformation between quantum and classical light can be realized by adjusting the driving detuning. This work studied quantum statistical characters of cavity photons in gravity coupled quadratical optomechanical system and showed the effects of gravity coupling on photon blockade. This should advance the development of manipulating photon blockade and has potential applications in quantum information science.

## Data Availability

All data generated or analysed during this study are included in this published article.

## References

[1] Birnbaum, K. M. *et al.* Photon blockade in an optical cavity with one trapped atom. *Nature* **436**(7047), 87–90 (2005). 16001065 10.1038/nature03804

[2] Chakram, S. *et al.* Multimode photon blockade. *Nat. Phys.* **18**(8), 879–884 (2022).

[3] Amazioug, M. *et al.* Strong photon antibunching effect in a double-cavity optomechanical system with intracavity squeezed light. *Quantum Inf. Process.* **22**(8), 301 (2023).

[4] Rabl, P. Photon blockade effect in optomechanical systems. *Phys. Rev. Lett.* **107**(6), 063601 (2011). 21902322 10.1103/PhysRevLett.107.063601

[5] Liao, J. Q. & Nori, F. Photon blockade in quadratically coupled optomechanical systems. *Phys. Rev. A* **88**(2), 023853 (2013).

[6] Leoński, W. & Tanaś, R. Possibility of producing the one-photon state in a kicked cavity with a nonlinear Kerr medium. *Phys. Rev. A* **49**(1), R20 (1994). 9910283 10.1103/physreva.49.r20

[7] Imamoglu, A. *et al.* Strongly interacting photons in a nonlinear cavity. *Phys. Rev. Lett.* **79**(8), 1467 (1997).

[8] Miranowicz, A. *et al.* Two-photon and three-photon blockades in driven nonlinear systems. *Phys. Rev. A* **87**(2), 023809 (2013).

[9] Majumdar, A. & Gerace, D. Single-photon blockade in doubly resonant nanocavities with second-order nonlinearity. *Phys. Rev. B* **87**(23), 235319 (2013).

[10] Sun, J. Y. & Shen, H. Z. Photon blockade in non-Hermitian optomechanical systems with nonreciprocal couplings. *Phys. Rev. A* **107**(4), 043715 (2023).

[11] Tian, L. & Carmichael, H. J. Quantum trajectory simulations of two-state behavior in an optical cavity containing one atom. *Phys. Rev. A* **46**(11), R6801 (1992). 9908087 10.1103/physreva.46.r6801

[12] Sarma, B. & Sarma, A. K. Single-photon blockade in a hybrid cavity-optomechanical system via third-order nonlinearity. *J. Phys. B At. Mol. Opt. Phys.* **51**(7), 075505 (2018).

[13] Hartmann, M. J., Brandao, F. G. S. L. & Plenio, M. B. Quantum many-body phenomena in coupled cavity arrays. *Laser Photon. Rev.* **2**(6), 527–556 (2008).

[14] Fink, J. M. *et al.* Observation of the photon-blockade breakdown phase transition. *Phys. Rev. X* **7**(1), 011012 (2017).

[15] Snijders, H. J. *et al.* Observation of the unconventional photon blockade. *Phys. Rev. Lett.* **121**(4), 043601 (2018). 30095925 10.1103/PhysRevLett.121.043601

[16] Lang, C. *et al.* Observation of resonant photon blockade at microwave frequencies using correlation function measurements. *Phys. Rev. Lett.* **106**(24), 243601 (2011). 21770569 10.1103/PhysRevLett.106.243601

[17] Deng, W. W., Li, G. X. & Qin, H. Photon blockade via quantum interference in a strong coupling qubit-cavity system. *Opt. Express* **25**(6), 6767–6783 (2017). 28381020 10.1364/OE.25.006767

[18] Huang, R. *et al.* Nonreciprocal photon blockade. *Phys. Rev. Lett.* **121**(15), 153601 (2018). 30362805 10.1103/PhysRevLett.121.153601

[19] Xie, H. *et al.* Nonreciprocal photon blockade in cavity optomagnonics. *Phys. Rev. A* **106**(5), 053707 (2022).

[20] Zhang, W. *et al.* Nonreciprocal photon blockade in a spinning resonator coupled to two two-level atoms. *Sci. China Phys. Mech. Astron.* **66**(4), 240313 (2023).

[21] Liu, Y. M. *et al.* Nonreciprocal photon blockade in a spinning optomechanical system with nonreciprocal coupling. *Opt. Express* **31**(8), 12847–12864 (2023). 37157436 10.1364/OE.486102

[22] Zhang, H. & Duan, Z. Photon blockade in the Jaynes-Cummings model with two-photon dissipation. *Opt. Express* **31**(14), 22580–22593 (2023). 37475365 10.1364/OE.492302

[23] Zheng, C. M. *et al.* Simultaneously enhanced photon blockades in two microwave cavities via driving a giant atom. *New J. Phys.* **25**(4), 043030 (2023).

[24] Feng, L. J., Ni, J. & Gong, S. Q. Photon blockade induced by two-photon absorption in cavity quantum electrodynamics. *Opt. Express* **32**(4), 5117–5130 (2024). 38439246 10.1364/OE.507086

[25] Xu, X. W. & Li, Y. Tunable photon statistics in weakly nonlinear photonic molecules. *Phys. Rev. A* **90**(4), 043822 (2014).

[26] Shi, H. Q., Xu, X. W. & Liu, N. H. Phonon blockade in a nanomechanical resonator quadratically coupled to a two-level system. *Sci. Rep.* **9**(1), 8754 (2019). 31217498 10.1038/s41598-019-45027-z PMC6584673

[27] Barzanjeh, S. *et al.* Optomechanics for quantum technologies. *Nat. Phys.* **18**(1), 15–24 (2022).

[28] Singh, S. K. & Ooi, C. H. R. Quantum correlations of quadratic optomechanical oscillator. *JOSA B* **31**(10), 2390–2398 (2014).

[29] Liao, J. Q. & Tian, L. Macroscopic quantum superposition in cavity optomechanics. *Phys. Rev. Lett.* **116**(16), 163602 (2016). 27152802 10.1103/PhysRevLett.116.163602

[30] Xie, H. *et al.* Macroscopic superposition states of a mechanical oscillator in an optomechanical system with quadratic coupling. *Phys. Rev. A* **100**(3), 033803 (2019).

[31] Zhu, B., Zhang, K. & Zhang, W. Optomechanical preparation of photon number-squeezed states with a pair of thermal reservoirs of opposite temperatures. *Photon. Res.* **11**(9), A26–A34 (2023).

[32] Singh, S. K., Asjad, M. & Ooi, C. H. R. Tunable optical response in a hybrid quadratic optomechanical system coupled with single semiconductor quantum well. *Quantum Inf. Process.* **21**(2), 47 (2022).

[33] Ghobadi, R. *et al.* Optomechanical micro-macro entanglement. *Phys. Rev. Lett.* **112**(8), 080503 (2014).

[34] Jiao, Y. F. *et al.* Nonreciprocal optomechanical entanglement against backscattering losses. *Phys. Rev. Lett.* **125**(14), 143605 (2020). 33064545 10.1103/PhysRevLett.125.143605

[35] McConnell, P. *et al.* Unconditional Wigner-negative mechanical entanglement with linear-and-quadratic optomechanical interactions. *Phys. Rev. A* **109**(3), 033508 (2024).

[36] Singh, S. K. *et al.* Entanglement and coherence in a hybrid Laguerre-Gaussian rotating cavity optomechanical system with two-level atoms. *J. Phys. B At. Mol. Opt. Phys.* **54**(21), 215502 (2021).

[37] Leoński, W. & Miranowicz, A. Kerr nonlinear coupler and entanglement. *J. Opt. B Quantum Semiclassical Opt.* **6**(3), S37 (2004).

[38] Fang, K. *et al.* Optical transduction and routing of microwave phonons in cavity-optomechanical circuits. *Nat. Photon.* **10**(7), 489–496 (2016).

[39] Li, J. *et al.* All-optical synchronization of remote optomechanical systems. *Phys. Rev. Lett.* **129**(6), 063605 (2022). 36018662 10.1103/PhysRevLett.129.063605

[40] Xia, Y. *et al.* Entanglement-enhanced optomechanical sensing. *Nat. Photon.* **17**(6), 470–477 (2023).

[41] Liu, S. *et al.* Realization of a highly sensitive mass sensor in a quadratically coupled optomechanical system. *Phys. Rev. A* **99**(3), 033822 (2019).

[42] Xu, H. *et al.* Topological energy transfer in an optomechanical system with exceptional points. *Nature* **537**(7618), 80–83 (2016). 27454555 10.1038/nature18604

[43] Ren, H. *et al.* Topological phonon transport in an optomechanical system. *Nat. Commun.* **13**(1), 3476 (2022). 35715403 10.1038/s41467-022-30941-0 PMC9205990

[44] Sheng, J., Yang, C. & Wu, H. Nonequilibrium thermodynamics in cavity optomechanics. *Fundam. Res.* **3**(1), 75–86 (2023). 38933566 10.1016/j.fmre.2022.09.005 PMC11197698

[45] Paulino, P. J., Lesanovsky, I. & Carollo, F. Nonequilibrium thermodynamics and power generation in open quantum optomechanical systems. *Phys. Rev. A* **108**(2), 023516 (2023).

[46] Ragole, S. *et al.* Thermodynamic limits for optomechanical systems with conservative potentials. *Phys. Rev. B* **96**(18), 184106 (2017).

[47] Braginsky, V. B. & Khalili, F. Y. *Quantum Measurement* (Cambridge University Press, Cambridge, 1995).

[48] Zhou, Y. H. *et al.* Spectrometric detection of weak forces in cavity optomechanics. *Opt. Express* **28**(19), 28620–28634 (2020). 32988129 10.1364/OE.398161

[49] Zhang, W. Z. *et al.* Optomechanical force sensor in a non-Markovian regime. *New J. Phys.* **19**(8), 083022 (2017).

[50] Cosco, F., Pedernales, J. S. & Plenio, M. B. Enhanced force sensitivity and entanglement in periodically driven optomechanics. *Phys. Rev. A* **103**(6), L061501 (2021).

[51] Yan, Z. F., He, B. & Lin, Q. Force sensing with an optomechanical system at room temperature. *Phys. Rev. A* **107**(1), 013529 (2023).

[52] Bemani, F. *et al.* Force sensing in an optomechanical system with feedback-controlled in-loop light. *Phys. Rev. Appl.* **17**(3), 034020 (2022).

[53] Zhao, W. *et al.* Weak-force sensing with squeezed optomechanics. *Sci. China Phys. Mech. Astron.* **63**(2), 224211 (2020).

[54] Vitali, D., Mancini, S. & Tombesi, P. Optomechanical scheme for the detection of weak impulsive forces. *Phys. Rev. A* **64**(5), 051401 (2001).

[55] Aldana, S., Bruder, C. & Nunnenkamp, A. Detection of weak forces based on noise-activated switching in bistable optomechanical systems. *Phys. Rev. A* **90**(6), 063810 (2014).

[56] Thompson, J. D. *et al.* Strong dispersive coupling of a high-finesse cavity to a micromechanical membrane. *Nature* **452**(7183), 72–75 (2008). 18322530 10.1038/nature06715

[57] Aspelmeyer, M., Kippenberg, T. J. & Marquardt, F. Cavity optomechanics. *Rev. Mod. Phys.* **86**(4), 1391 (2014).

[58] Nunnenkamp, A. *et al.* Cooling and squeezing via quadratic optomechanical coupling. *Phys. Rev. A* **82**(2), 021806 (2010).

[59] Qvarfort, S. *et al.* Gravimetry through non-linear optomechanics. *Nat. Commun.* **9**(1), 3690 (2018). 30206216 10.1038/s41467-018-06037-z PMC6133990

[60] Biswas, D. *et al.* Gravitational optomechanics: Photon-matter entanglement via graviton exchange. *Phys. Rev. D* **108**(6), 064023 (2023).

[61] Qvarfort, S. *et al.* Optimal estimation of time-dependent gravitational fields with quantum optomechanical systems. *Phys. Rev. Res.* **3**(1), 013159 (2021).

[62] Qvarfort, S., Rätzel, D. & Stopyra, S. Constraining modified gravity with quantum optomechanics. *New J. Phys.* **24**(3), 033009 (2022).

[63] Scala, M. *et al.* Matter-wave interferometry of a levitated thermal nano-oscillator induced and probed by a spin. *Phys. Rev. Lett.* **111**(18), 180403 (2013). 24237492 10.1103/PhysRevLett.111.180403

[64] Mo, K. B., Jo, T. G. & Dahl, J. P. Displaced squeezed number states: Position space representation, inner product, and some applications. *Phys. Rev. A* **54**(6), 5378 (1996). 9914108 10.1103/physreva.54.5378

[65] Kowalewska-Kudłaszyk, A. *et al.* Two-photon blockade and photon-induced tunneling generated by squeezing. *Phys. Rev. A* **100**(5), 053857 (2019).

[66] Bin, Q., Lü, X. Y., Bin, S. W. & Wu, Y. Two-photon blockade in a cascaded cavity-quantum-electrodynamics system. *Phys. Rev. A* **98**, 043858 (2018).

[67] Radulaski, M., Fischer, K. A., Lagoudakis, K. G., Zhang, J. L. & Vučković, J. Photon blockade in two-emitter-cavity systems. *Phys. Rev. A* **96**, 011801 (2017).

